# Definite Implant Position as Novel Readout for Effectiveness of Ridge Preservation Indicates to Beneficial Effect of Combined Treatment with Platelet-Rich Fibrin (PRF) and Xenogenic Biomaterial in Bone Regeneration

**DOI:** 10.3390/bioengineering13060710

**Published:** 2026-06-20

**Authors:** Anja Heselich, Sebastian Mann, Jörg-Ulf Wiegner, Shahram Ghanaati

**Affiliations:** 1 FORM-Lab, Frankfurt Orofacial Regenerative Medicine, Department for Oral, Cranio-Maxillofacial and Facial Plastic Surgery, Medical Center of the Goethe University Frankfurt, Goethe University, 60590 Frankfurt am Main, Germany; 2 Saalepraxis, Saalstraße 35, 07318 Saalfeld/Saale, Germany

**Keywords:** dental implants, biomaterials, ridge preservation, platelet-rich fibrin PRF, CBCT, image-analysis

## Abstract

Methods of ridge preservation following tooth extraction, aiming to maintain alveolar bone volume and support tissue regeneration, have been extensively researched. Continuously, new approaches and materials are being explored in this context. To scientifically evaluate outcomes, the pre-implant situation is usually assessed radiologically, histologically, and/or clinically. However, the influence of ridge preservation on implant placement itself is rarely examined in depth, and if at all, the focus has been on implant stability or survival rates. Based on the assumption that preoperative radiological assessment, including cone beam computed tomography, provides only an indirect and inherently limited approximation of actual intraoperative bone condition, undetected factors such as insufficient bone density, mechanically unfavorable trabecular structure, or incompletely resorbed residual biomaterial may necessitate a shift of the implant from the preferred position originally occupied by the tooth root. We therefore established a method that evaluates and categorizes implant position in three dimensions based on radiological data post-implantation. Our data, derived from a multicenter randomized clinical trial (RCT), demonstrate that the greatest positional deviations are observed without preservation, whereas the combination of biomaterial and PRF most frequently allowed for central implant placement. The proposed method proves well suited for evaluating the outcome of ridge preservation procedures. The findings demonstrate that both the absence and presence, and further the type, of preservation have a measurable influence on the final implant positioning.

## 1. Introduction

It is well known that the alveolar process is prone to remodeling dynamics upon regeneration after tooth loss. In healthy dentins, the teeth are anchored within the bone of the alveolar process by Sharpey’s fibers [1], and under physiological conditions, the bone undergoes continuous resorptive and formative remodeling to maintain its structural integrity [2,3]. This remodeling is influenced by multiple factors, particularly mechanical loading [4,5,6]. It is assumed that mechanosensitive cells detect strain and thereby regulate osteoblast and osteoclast activity [7]. When a tooth is absent, this mechanical stimulus is lost and bone resorption follows. On average, after six months, a vertical bone loss of 11% to 22% and a horizontal bone loss of 29% to 63% can be observed [8].

Following tooth extraction, a blood clot forms within the alveolus, marking the first three days of wound healing [9]. This clot achieves hemostasis and provides a scaffold for cells and growth factors involved in regeneration, including platelet-derived growth factor (PDGF), vascular endothelial growth factor (VEGF), and transforming growth factor beta (TGF-β). The subsequent inflammatory phase can be divided into an early and a late stage. Neutrophil granulocytes predominate initially, followed by macrophages, both contributing to infection control within the wound. During the proliferative phase, fibroblasts migrate along gradients of TGF-β and PDGF. They synthesize proteoglycans, fibronectin, and procollagen types I and III [10]. In the final modeling and remodeling phase, previously formed woven bone is gradually replaced by lamellar bone [11]. In humans, this entire process extends over several months and may last years [10]. The course varies considerably between individuals, which renders wound healing difficult to predict [12].

To limit resorptive changes in the alveolar bone, clinical alveolar ridge preservation procedures can be performed [13]. For such, autologous, allogeneic, xenogeneic, and alloplastic materials are available for defect augmentation [14,15,16], and additionally, or alternatively, autologous blood concentrates may be applied as well [16,17].

Autologous graft material, harvested from the patient’s own bone, would probably present the most promising material for its excellent biocompatibility and low risk of adverse effects due to its autologous origin. Further, it introduces viable cells directly into the wound environment [18], whereas allogeneic bone, which originates from donor tissue, carries a potential risk of immune reaction or infection transmission [19,20]. Xenogeneic bone substitutes consist of inorganic particles derived from animal sources and lack biological active components. The risk of immunologic response is low. These materials exhibit osteoconductive properties. They stabilize the blood clot and prevent its detachment from the alveolar wall. In addition, they provide a scaffold that supports physiological bone regeneration [16,19,21]. Alloplastic materials are synthetically manufactured materials without biological active components and therefore show predominately osteoconductive characteristics. However, due to sterile production protocols, the risk of disease transmission is generally negligible [19,22]. In addition to osseous substitutes, xenogeneic soft tissue replacement materials, such as collagen membranes, are commonly employed as barrier membranes to cover bone substitutes in guided bone regeneration procedures. Their biocompatibility and ability to interact with the tissue environment support proper healing and may additionally influence the efficacy of ridge preservation and defect regeneration [23].

An alternative to conventional grafting materials is autologous blood concentrates like platelet-rich fibrin (PRF) [24], which represents a further development of platelet-rich plasma (PRP) [25]. For PRF preparation, venous blood is drawn from the patient and centrifuged in a one-step approach without the use of anticoagulants. The resulting product is therefore entirely autologous [25]. After centrifugation, three layers become visible within the tube. Erythrocytes accumulate at the bottom. The PRF clot is located centrally and is surrounded by acellular plasma [26]. PRF contains growth factors such as PDGF, TGF-β1, insulin-like growth factor 1 (IGF-1) [27], VEGF, and interleukins 1β, 4, and 6 [26]. During matrix remodeling, cytokines are gradually released from the fibrin scaffold, which further promotes wound healing [27]. The fibrin matrix itself contributes to several beneficial clinical effects. Its three-dimensional architecture enhances angiogenesis [28], and further, fibrin has been shown to play a role in immune defense [29]. The resulting fibrin network of the autologous blood concentrate PRF facilitates entrapment and accumulation of mesenchymal stem cells [25].

PRF has been extensively investigated since its initial development, and its preparation protocols have been continuously refined to further enhance its performance. One of these approaches is known as the low-speed centrifugation concept, LSCC, where PRF is produced using reduced centrifugal force and centrifugation time [30,31]. Low-speed variants thereby demonstrate higher concentrations of growth factors such as PDGF, TGF-β1, IGF-1, epidermal growth factor, and VEGF compared to standard PRF [30]. The low-speed centrifugation concept also led to the development of injectable PRF (I-PRF), resulting in a liquid PRF fraction that can be injected or combined with bone substitute materials [32,33].

The aim of socket or ridge preservation procedures post-extraction is usually to provide a sufficient bone structure to allow for insertion of stable and long-lasting dental implants. The implant placement following extraction thereby requires an optimal and physiologically preserved alveolar ridge to protect adjacent anatomical structures. In the mandible, a minimum safety distance of 2 mm from the inferior alveolar nerve should be maintained [34]. In the maxilla, perforation of the nasal floor or the Schneiderian membrane of the maxillary sinus must be avoided [35,36]. Dental spacing guidelines are equally important. The distance to the root of an adjacent tooth should be at least 1.5 mm [37]. The implant shoulder should also maintain a distance of 1.5 mm to 2 mm from the neighboring tooth to prevent future abutment complications [38]. When placing two implants adjacent to one another, an inter-implant distance of at least 3 mm is recommended [39]. Excessive spacing should also be avoided, as it may lead to unfavorable load distribution, secondary bone loss, and compromised esthetics [38]. The implant should be positioned centrally within the edentulous space. This principle applies to the mesiodistal dimension, where the implant should be aligned parallel to the roots of adjacent teeth. In the buccolingual dimension, central positioning within the alveolar ridge is likewise preferable. If this is not feasible, the implant should be surrounded by at least 1.5 mm to 2 mm of buccal bone and at least 0.5 mm of oral bone [40]. Insufficient bone coverage may result in recession, esthetic impairment, food impaction, and biomechanical overload. Vertical positioning is commonly referenced to the cemento-enamel junction and the implant shoulder and recommended vertical distances of 3 mm [41].

To achieve the aforementioned bone dimensions deemed to be a valuable basis for long-term implantation success, the aim remains for optimization of bone regeneration. One approach is the biological enhancement of inherently inert bone substitute materials through the addition of an autologous preparation such as PRF, aiming to combine structural stability with biological activity. The primary objective of our clinical trial was therefore to compare a bovine-derived xenogeneic bone substitute material (BSM) containing 10% collagen, Bio-Oss Collagen^®^ (Geistlich Pharma AG, Wolhusen, Switzerland), with the same material combined with liquid low-PRF, generated using the LSSC-concept [31]. These groups were further compared with sites that received no ridge preservation and with sites filled exclusively with solid high-speed PRF (LSCC) [31].

Scientific evaluation of ridge preservation materials can be performed clinically, histologically, or radiographically. Preoperative radiological assessment, including CBCTs, provides only an indirect approximation of the actual bone conditions at the implant site [42]. Crucially, any CBCT-derived grayscale values are subject to considerable variability arising from scanner-specific factors such as field-of-view limitations, scatter radiation, and reconstruction algorithms, which preclude their reliable use as quantitative measures of bone density [43,44]. These limitations can have a direct impact on radiological evaluations of previously augmented sites. Residual biomaterial particles cannot be reliably distinguished from newly formed bone or unmineralized tissue on radiological imaging alone, and metal or ceramic artifacts in close proximity may further obscure local bone structures [44].

Therefore, the intraoperative bone quality can, despite a good preoperative imaging-based prediction, prove to be insufficient in terms of cortical density, mechanically unfavorable trabecular structure, or incompletely resorbed and residual graft material. This systematic gap between radiological prediction and intraoperative reality can necessitate a repositioning of the implant away from the tooth-root-derived preferred position. Taking the mentioned aspects into consideration, we propose the hypothesis that implant position is potentially dependent on the efficacy of the selected ridge preservation procedure with respect to bone preservation and regeneration. We therefore established a method that evaluates and categorizes implant position in three dimensions based on post-implantation radiological data.

The radiographic categorization of final implant position we described here represents a novel method for assessing the effectiveness of different therapeutic approaches. It is simple to perform, does not require baseline radiographs, and can be implemented efficiently in daily clinical practice. By focusing directly on the conditions present at the time of implantation, this method evaluates precisely the outcome that ridge preservation therapy seeks to achieve and therefore provides a clinically relevant assessment of the true effectiveness of the materials or preservation technique used.

## 2. Materials and Methods

### 2.1. Clinical Trial

#### 2.1.1. Study Design

The data described here are part of a multicenter, prospective, parallel-arm randomized controlled clinical IIT trial. The trial is registered at the German Clinical Trials Register (Deutsches Register Klinischer Studien, DRKS) under registration number DRKS00024023.

#### 2.1.2. Ethical Approval

The clinical study analyzed here was approved by the Institutional Review Board of the Ethical Committee of the Medical Department of Goethe University (IRB approval #20-879). The study followed the Declaration of Helsinki and national regulations in Germany for human studies. All participants provided written informed consent after being informed of the study’s procedures and objectives.

#### 2.1.3. Inclusion and Exclusion Criteria

Patients (≥18, ≤98 years) requiring premolar or molar extractions (excluding 3rd molars) and planned for dental implant therapy were recruited. Additionally, candidates must have an indication for socket or ridge preservation and be suitable for a two-stage intervention. Participants were expected to adhere to study-related requirements and guidelines.

Exclusion criteria included severe periodontal disease or acute pericoronitis affecting the remaining dentition, mucosal disease in the treatment areas, ongoing antibiotic treatment, previous augmentation procedures in the defect region, uncontrolled diabetes, alcohol abuse, and inability to follow post-surgery instructions.

#### 2.1.4. Randomization

Patients were randomly assigned to either of the four groups (Control Group 1: No Preservation; Control Group 2: high-RCF PRF; Test Group 1: Bio-Oss Collagen^®^; Test Group 2: Bio-Oss Collagen^®^ + low-RCF PRF). Based on the sample size calculation, computer-generated randomization (GraphPad Software (Version 10.6.1), LLC, Boston, MA, USA) was performed by a study member not involved in patients‘ treatment. Each patient was allocated to a sealed randomization letter, opened by the surgeon immediately before extraction.

#### 2.1.5. Follow-Up and Outcome Measures

Tooth extraction and treatment were assessed for bone condition and regeneration after tooth extraction and after a regeneration time of three months. The primary outcome was bone regeneration and preservation three months after extraction.

### 2.2. Surgical Procedures

#### 2.2.1. Tooth Extraction and Alveolar Ridge Preservation

The Extractions were performed under local anesthesia. Sockets were inspected, curetted, and rinsed with sterile 0.9% saline. In the alveolar ridge preservation groups, sockets were either filled with bone substitute material (BSM; Bio-Oss Collagen^®^, Geistlich Pharma AG, Wolhusen, Switzerland; Test Group 1) or with the BSM in combination w/low-RCF PRF (Test Group 2). In all groups, sockets were then sutured tension-free with non-resorbable horizontal mattress sutures. Control sockets remained untreated (Control Group 1) or filled with solid high-RCF PRF (Control Group 2), but received identical suturing in the BSM groups.

#### 2.2.2. PRF Preparation

Autologous platelet-rich fibrin (PRF) was prepared according to the Low Speed Centrifugation Concept (LSCC), as previously described elsewhere [24]. For patients within the PRF treatment groups, the patient’s peripheral blood was collected in special PRF blood vacuum sampling tube(s) without any additives (Process for PRF, Nice, France). Filled sampling tubes were centrifuged in a PRF-Duo Quattro medical device centrifuge for PRF (Process for PRF, Nice, France). All tubes were centrifuged within 3 min of collection.

Solid high-RCF PRF was gained at 2400 rpm (710× *g*) for 8 min. It was harvested by carefully removing the coagulated PRF clot using sterile tweezers. The attached residual red blood cell phase was carefully scraped off with the blunt side of sterile scissors and the remaining solid PRF clot was pressed into a solid PRF plug for direct application into an extraction socket. For combination with bone substitute material, low-RCF PRF, centrifuged at 600 rpm (44× *g*) for 8 min, was prepared and mixed with the BSM prior to application.

#### 2.2.3. Follow-Up

Suture removal was performed approx. 10 days after extraction surgery. After 3 months of regeneration time, implantation surgery was planned and performed, followed again by suture removal approx. 10 days later. Final prosthetics was usually applied within the next 3 months. Final follow-up with close-out was performed 9 months post-extraction. All timepoints were defined and conducted in accordance with the approved Clinical Investigation Protocol.

### 2.3. Radiological Evaluations

#### 2.3.1. Software and Data Format

The imaging software Fiji (Fiji Is Just ImageJ; v2.16.0) served as the foundation for the image-based evaluation of mineralized bone volume. Fiji is built on ImageJ2, an advanced version of the scientific and medical image analysis software ImageJ provided by the National Institutes of Health (NIH, Bethesta, MD, USA; imagej.nih.gov) [45]. All processing and analyzing tools used and implemented in the analysis macro are included in Fiji.

3D Cone Beam Computed Tomography (CBCT) images used for analysis had been recorded and analyzed in DICOM format.

#### 2.3.2. Radiographic Image Preparation

**Figure 1:**
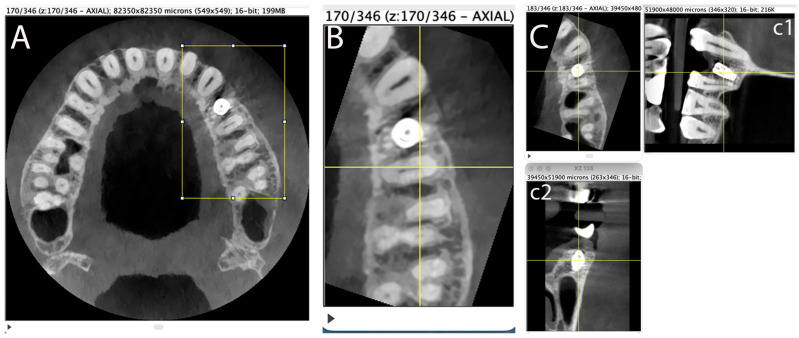
Example image for DICOM-stack preparation for further evaluation. (**A**): Imported stack, with identified region of interest ROI (yellow rectangle), (**B**): cropped and rotated ROI, and (**C**): rotated ROI with additional orthogonal view in sagittal (**c1**) and frontal (**c2**) perspective (yellow cross-lines mark the selected position).

The datasets provided in DICOM format were imported as cohesive datasets using Fiji’s “Bio-Formats Importer”. DICOM stacks were then reduced to the image slices from the apical region of the alveolus to the crown tips of adjacent teeth. Then, an area representing only the alveolus and one to two adjacent teeth was cropped. Finally, the resulting image stack was rotated so that the alveolus and adjacent teeth were aligned vertically (see Figure 1).

#### 2.3.3. Evaluation of Implant Position

**Figure 2:**
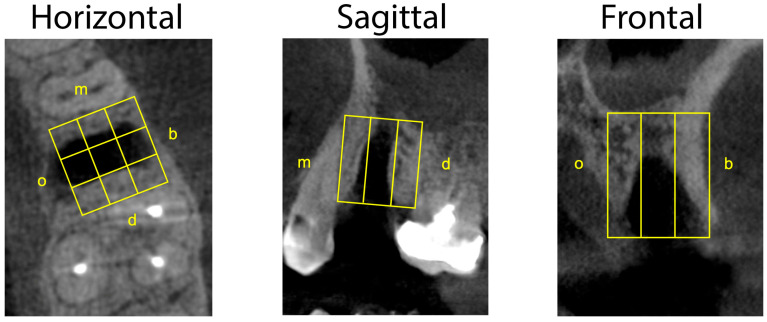
Schematic illustration of possible implant positions around the center of the former extraction socket in horizontal, sagittal, and frontal views. Abbreviations: m = mesial, d = distal, o = oral, and b = buccal.

After preparation of the rotated DICOM-stack, including the ROI, the jaw is displayed in the horizontal cross-sectional plane. However, for a complete three-dimensional assessment, the sagittal and frontal planes are additionally required. This can be done via Fiji’s “Orthogonal Views” function, which renders the missing planes visible (see Figure 1C). The XY-plane corresponds to the frontal plane, while the YZ-plane represents the sagittal plane. The movable crosshair is then positioned centrally over the implant at crestal height in order to evaluate the implant position in relation to the surrounding bone and adjacent teeth across all planes based on a predefined grid, as shown in Figure 2.

**Figure 3:**
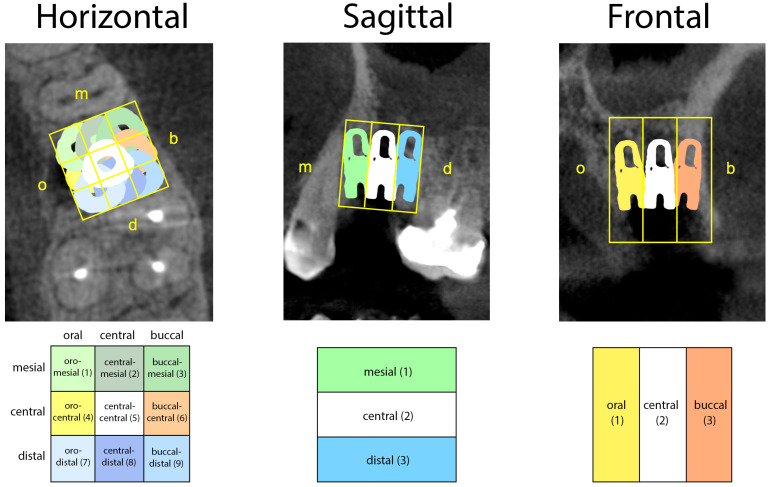
Schematic illustration of the categorization of implant positions based on implant placement around the center of the former extraction socket in horizontal, sagittal, and frontal views. (**Left panel**): implants can be placed central or shifted in mesial or distal, and/or oral or buccal direction, resulting in nine possible positions in occlusal view. (**Middle panel**): in sagittal view, observation of shifts from the central position is possible in mesial or distal direction. (**Right panel**): in frontal view, implant position can be categorized based on shifts in oral or buccal direction. Abbreviations: m = mesial, d = distal, o = oral, and b = buccal.

The classification according to the proposed grid (see Figure 2) in mesial-to-distal and oral-to-buccal orientation yields eight additional positions in horizontal view beyond the optimal, central position: the implant may be placed too far mesially, distally, buccally, or orally. In addition, intermediate positions exist between these four locations, namely oro-mesial, buccal–mesial, oro-distal, and buccal–distal positioning. This results in a grid into which the implant position can be systematically categorized (Figure 3, left panel).

In the sagittal plane, the implant may be centrally positioned or displaced too far in the mesial or distal direction, yielding a classification into three distinct zones (Figure 3, middle panel). Similarly, in the frontal plane, the implant may be centered or positioned too far orally or buccally (Figure 3, right panel). For this assessment, the apical region was examined, and an equivalent three-zone classification was applied. Following this scheme, each implant position was visually determined and assigned to the corresponding category (Figure 3).

#### 2.3.4. Implant Position Shifts in Frontal View

For evaluation of reproducibility of descriptive analysis of the implant positioning, 20 cases in total (n = 10 per tooth type) were evaluated independently by an experienced rater and an inexperienced one. Cohen’s Kappa analysis of inter-rater deviation was performed using R statistical software (Version R 4.6.0 GUI 1.83 High Sierra build (8601) at a confidence interval of 95%).

### 2.4. Statistics, Sample Size, and Included Data Sets

Statistical analysis was performed using the software GraphPad Prism (Version 10.6.1 for Windows, GraphPad Software, LLC, Boston, MA, USA).

Sample size calculation for the main trial on which this analysis is based was performed as follows: Based on prior experience, an expected percentage range of 32–76% for the primary endpoint of mean bone regeneration and an effect size of up to d = 1.2 were assumed. Assuming d = 1.2, a sample size of 19 cases per group (eight groups, i.e., four treatment groups × two tooth groups), not accounting for drop-outs, was required to achieve 80% statistical power at a Bonferroni-corrected significance level of α = 0.833% (two-sided α = 5% divided by six pairwise comparisons between four treatment groups).

**Figure 4:**
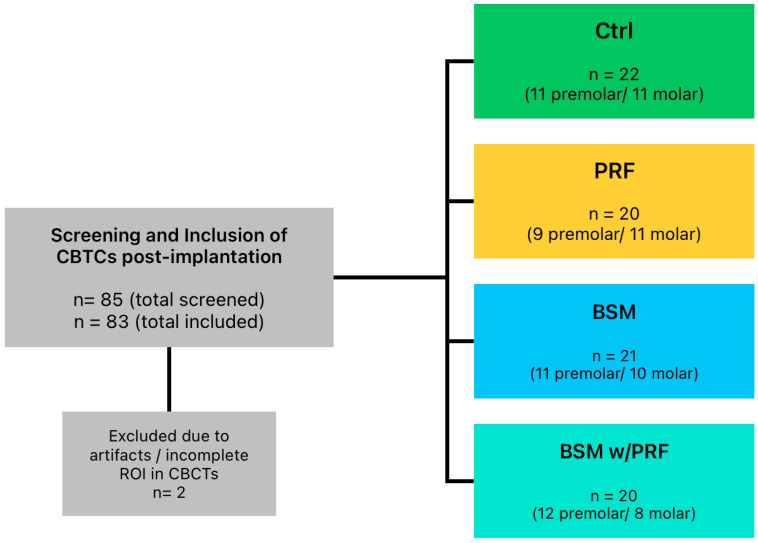
Distribution of cases included for evaluation of implant position according to group and tooth type.

Within the clinical trial, 83 CBCTs post-implantation were available for preliminary analysis of implant position evaluation: n = 22 (11 premolar/11 molar) for control group, n = 20 (9 premolar/11 molar) for PRF group, n = 21 (11 premolar/10 molar) for BSM group, and n = 20 (12 premolar/8 molar) for BSM w/PRF group (Figure 4).

The distributions per group to the individual implant positions in horizontal, sagittal and frontal views were calculated as percentages per group and visualized as heatmaps.

## 3. Results

### 3.1. Tooth Distribution and Demographics

A total of 83 of 85 extraction sockets were included: n = 22 (11 premolar/11 molar) for control group, n = 20 (9 premolar/11 molar) for PRF group, n = 21 (11 premolar/10 molar) for BSM group, and n = 20 (12 premolar/8 molar) for BSM w/PRF group. Two sockets were excluded due to artifacts or incomplete ROI (Figure 4). The age and sex distribution showed an overall homogeneous distribution for all patients and within groups. Overall mean age was 55.96 years in all patients, with 49.4% male and 50.6% female patients.

Mean age in the control group was 55.94 years, in the PRF group 54.15 years, in the BSM group 54.71 years, and in the BSM w/PRF group 59.10 years.

Distribution according to sex per group showed n = 12 male and n = 10 female patients for control group, n = 10 male and female each for PRF group, n = 10 male and n = 11 female patients for BSM group, and n = 9 male and n = 11 female patients for BSM w/PRF group.

### 3.2. Evaluation of Implant Position

**Figure 5:**
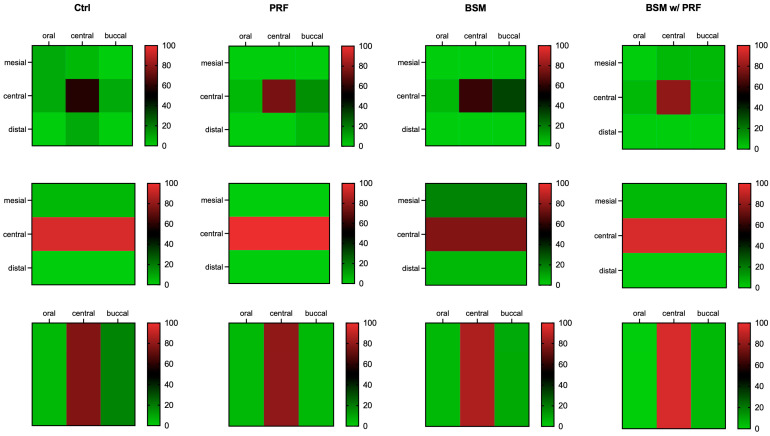
Distribution of implant position in horizontal (**upper panel**), sagittal (**middle panel**), and frontal (**lower panel**) views. Deviations from the favorable central position were observed in all groups and evaluation planes. Highest fraction of centrally positioned implants was observed in the BSM w/PRF group (most right column). Data are presented as heatmaps with percentage distribution of all cases within each group; n = 22 (11 premolar/11 molar) for control group, n = 20 (9 premolar/11 molar) for PRF group, n = 21 (11 premolar/10 molar) for BSM group, and n = 20 (12 premolar/8 molar) for BSM w/PRF group (see also Table 1, Table 2 and Table 3). Legend: color range from bright green (=0%) to bright red (=100%), black equaling 50%.

Effectiveness of ridge preservation was evaluated by categorization of implant position in horizontal, sagittal and frontal perspectives, as described in Section 2.3.3. Resulting distribution of implant positions was transferred to heat maps for visualizing differences between the four analyzed groups (Figure 5).

**Table 1:** Implant position distribution in the horizonal plane according to tooth type, premolar or molar, for each group. Data are shown as percent, 100% = total per group and tooth type (premolar/molar/overall distribution (=molar and premolar pooled)). n = 22 (11 premolar/11 molar) for control group, n = 20 (9 premolar/11 molar) for PRF group, n = 21 (11 premolar/10 molar) for BSM group, and n = 20 (12 premolar/8 molar) for BSM w/PRF group.

	Premolar				Molar				Overall Distribution			
Implant Position	**Ctrl**	**PRF**	**BSM**	**BSM w/PRF**	**Ctrl**	**PRF**	**BSM**	**BSM w/PRF**	**Ctrl**	**PRF**	**BSM**	**BSM w/PRF**
oro-mesial	18%	0	0	0	0	0	0	0	9%	0	0	0
central–mesial	0	0	0	8%	9%	0	0	0	5%	0	0	5%
buccal–mesial	0	0	0	0	0	0	0	13%	0	0	0	5%
oro-central	0	11%	0	0	18%	0	10%	13%	9%	5%	5%	5%
central–central	64%	78%	55%	92%	55%	73%	70%	63%	59%	75%	62%	80%
buccal–central	9%	11%	45%	0	9%	18%	20%	13%	9%	15%	33%	5%
oral–distal	0	0	0	0	0	0	0	0	0	0	0	0
central–distal	9	0	0	0	9%	0	0	0	9%	0	0	0
buccal–distal	0	0	0	0	0	9%	0	0	0	5%	0	0

**Table 2:** Implant position distribution in sagittal view according to tooth type, premolar or molar, for each group. Data are shown as percent, 100% = total per group and tooth type (premolar/molar/overall distribution (=molar and premolar pooled)). n = 22 (11 premolar/11 molar) for control group, n = 20 (9 premolar/11 molar) for PRF group, n = 21 (11 premolar/10 molar) for BSM group, and n = 20 (12 premolar/8 molar) for BSM w/PRF group.

	Premolar				Molar				Overall Distribution			
Implant Position	**Ctrl**	**PRF**	**BSM**	**BSM w/PRF**	**Ctrl**	**PRF**	**BSM**	**BSM w/PRF**	**Ctrl**	**PRF**	**BSM**	**BSM w/PRF**
mesial	27%	0	9%	0	9%	0	0	13%	18%	0	5%	5%
central	73%	100%	91%	100%	82%	100%	100%	88%	77%	100%	95%	95%
distal	0	0	0	0	9%	0	0	0	5%	0	0	0

**Table 3:** Implant position distribution in frontal view according to tooth type, premolar or molar, for each group. Data are shown as percent, 100% = total per group and tooth type (premolar/molar/overall distribution (=molar and premolar pooled)). n = 22 (11 premolar/11 molar) for control group, n = 20 (9 premolar/11 molar) for PRF group, n = 21 (11 premolar/10 molar) for BSM group, and n = 20 (12 premolar/8 molar) for BSM w/PRF group.

	Premolar				Molar				Overall Distribution			
Implant Position	**Ctrl**	**PRF**	**BSM**	**BSM w/PRF**	**Ctrl**	**PRF**	**BSM**	**BSM w/PRF**	**Ctrl**	**PRF**	**BSM**	**BSM w/PRF**
oral	0	22%	0	0	9%	9%	10%	0	5%	5%	5%	0
central	64%	67%	82%	92%	91%	91%	90%	100%	77%	80%	86%	95%
buccal	36%	11%	18%	8%	0	0	0	0	18%	5%	9%	5%

#### 3.2.1. Implant Position Shifts in Occlusal View

In the horizontal plane, observed in occlusal view (Figure 5, upper panel; Table 1), the central position usually represents the natural position of the former root and is therefore deemed to be the most favorable one. The analysis shows the highest proportion of implants (80%) placed in this favorable central position in the group where BSM was combined with PRF. The PRF group demonstrates 5% lower fraction, with 75% of implants within this group positioned centrally. The BSM group showed a rate of only 62% of implants placed centrally, whereas the control group without preservation showed the lowest rates of optimal positioning, with only 59%. Furthermore, in this group, a greater number of implants were shifted in central–distal (9%), oral–central (9%), and oral–mesial (9%) directions compared to the other groups. Offset placing was also observed in the central–mesial (5%) and vestibular–central (9%) directions within this group. In the BSM group, the predominant shift from the central position occurred in the buccal–central direction (33%), with a smaller proportion of implants displaced toward the oral–central region (5%). In the PRF group, deviation from the central position was most frequently observed in the buccal–central direction (15%), with fewer implants shifted toward the oral–central (5%) and buccal–distal (5%) positions. In the BSM w/PRF group, deviations were rare and evenly distributed across the oral–central (5%), central–mesial (5%), buccal–mesial (5%), and buccal–central (5%) directions.

Sub-analysis according to tooth type (Table 1) showed that deviation from the central position in horizontal view was often more pronounced in molar than in premolar former extraction sockets. In the control group, 64% of the premolar cases were placed centrally, 9% shifted to either buccal–central position or central–distal position, and 18% to oro-mesial position. The molar cases showed fewer cases in the central position (55%) and more cases with shifts to other directions (Table 1).

In the PRF group, implants were closely placed in central positions with 78% centrally and 11% each shifted more buccally or orally. In the respective molar cases, the majority as well was placed centrally (73%); however, the shift of the remaining cases from the central position was mostly buccally oriented (Table 1).

The BSM group showed almost equally distributed cases of premolar implants to central (55%) and buccal–central (45%) position. The respective molar cases could be placed more often centrally (70%), or with shifts to oral or buccal direction (Table 1).

The premolar group with the majority of cases in a favorable central position was the BSM w/PRF group, with 92% placed there and only 8% shifted to the central–mesial position. Regarding the molars, a more pronounced shift to other positions was observed, with only 63% in the central position (Table 1).

#### 3.2.2. Implant Position Shifts in Sagittal View

In sagittal (buccal to oral) view (Figure 5, middle panel; Table 2), the group treated with PRF alone shows the highest proportion of implants located in the central position compared to all other groups (100%), which was true for both tooth types (Table 2). The BSM group and the combination group of BSM w/PRF both demonstrate slight reductions, with identical distributions in the central zone (95% each), and in both groups, a small proportion of implants were shifted in the mesial direction (5% each). The control group without preservation, in contrast, presents an overall less favorable distribution. Here, the fewest implants were positioned centrally (77%), while a considerable proportion were shifted mesially (18%) and a smaller number distally (5%).

Sub-analysis according to tooth type showed the majority of shift in the control group, with only 73% of the premolar cases and 82% of the molar cases placed centrally (Table 2). While in the PRF group, all implants, independent of tooth type, were placed centrally, the BSM group showed differences between premolars and molars. All of the molar cases, but just 91% of premolar, of the BSM group were placed centrally (Table 2). The BSM w/PRF group showed an inverted pattern with all of the premolar cases, but only 88% of the molar cases were placed centrally (Table 2).

#### 3.2.3. Implant Position Shifts in Frontal View

In the frontal (mesial to distal) view (Figure 5 lower panel; Table 3), only minimal differences in the central position were observed across the four groups. Nevertheless, a gradual reduction is apparent, beginning with the BSM w/PRF group (95%), followed by the BSM group (86%), then the PRF group (80%), and finally the control group without preservation (77%). Accordingly, the control group shows the highest fraction of implants shifted buccally (18%) and orally (5%). In the PRF group, the shift was inverted, with several implants placed more orally (15%) and fewer more buccally (5%). In the BSM group, implants were similarly shifted orally (5%) and buccally (9%), though to a lesser overall extent than in the other groups. The fewest deviations from the central position were observed in the BSM w/PRF group, with only 5% of implants shifted buccally.

Further analysis of distribution according to tooth type showed most favorable positioning for all molar cases, with 91% each in control and PRF groups, 90% in the BSM group, and even 100% of the cases in the BSM w/PRF group (Table 3). The premolar cases showed clearly higher rates of shifts to oral or buccal position, with only 64% of control cases, 67% of PRF group cases, 82% of BSM group cases, and 92% of BSM w/PRF group cases in the central position (Table 3).

## 4. Discussion

A broad variety of analysis methods have been developed and recommended in the literature to achieve a deeper insight into which variant of material composition and/or socket or ridge preservation offers an advantage above others; even two from our own lab [46,47]. As scientific evaluation of ridge preservation materials can be performed clinically, histologically, or radiographically, the radiological option allows for non-invasive observation of the bony situation. Nevertheless, evaluation of the bone dimensions radiologically still cannot give a definite conclusion on the in vivo situation the clinician will find during surgery. Therefore, radiologically unobserved conditions, like insufficient soft density or loose residual material, can lead to necessary modification of planned implant positioning [48]. Considering scientific evaluations of clinical trial data, the risk remains that a potentially efficient preservation approach, based on radiological evaluation of the bone dimensions, is not supported by the real in vivo observation of the treatment outcome.

The radiographic categorization of final implant position we described here represents a novel method for assessing the effectiveness of different therapeutic approaches. By focusing directly on the conditions of the implant positioning, resulting from radiological planning and the finally observed real-life situation of the implant site, this method evaluates precisely the outcome that ridge preservation therapy seeks to achieve and therefore provides a clinically relevant assessment of the true effectiveness of the materials or preservation technique used. It should be noted that the proposed grid-based assessment of implant position is primarily intended as a systematic evaluation tool for comparative research on ridge preservation procedures rather than a method applicable to daily clinical routine.

With the newly developed method for determining the final implant position, clear differences between the test groups in our descriptive analysis of the controlled randomized clinical trial were revealed. In general, any form of alveolar ridge preservation resulted in better outcomes than no preservation, and the combination group applying BSM and PRF achieved the most favorable results. PRF alone yielded better results than BSM alone, whereas the control group of natural healing performed the worst.

In the literature, various methods that can also be applied either in addition to or exclusively after implant placement for evaluation are described. The analysis of bone density has long been used to compare extraction sockets over time [49,50] and continues to be refined [46]. However, once implants are placed, this assessment is no longer feasible. As an alternative, clinical measurement of implant stability can be employed [51,52,53]. Ultimately, implant stability reflects bone quality and density [54,55], but is also to a certain extent dependent on the implant type used [56]. With respect to our research question, determining the implant stability presents limitations, since no baseline value can be obtained immediately after preservation. Only outcomes from different procedures can be compared, for example, radiographic bone density and subsequent implant stability. In addition, retrospective comparisons between individuals must be considered imprecise due to the numerous factors influencing bone density [4,5,57,58].

Further studies that include implants in their evaluation frequently compare survival rates between test groups. This approach addresses the question of definitive clinical benefit using a straightforward and rapid method. However, clear conclusions can rarely be drawn [59,60,61,62,63,64,65]. One study reported that the percentage difference in implant survival between the use of bone substitute material (BSM) Bio-Oss^®^ and the control group did not exceed 1.5% [63]. In line with our findings, ridge preservation tended to perform better in that investigation [63]. In contrast, another study describing implant survival after one year reached the opposite conclusion, reporting survival rates of 95.2% following ridge preservation and 100% after physiological healing [61]. Overall, consideration of survival rates suggests that a favorable long-term prognosis for implants can be expected regardless of the therapy performed [59,60,61,62,63,64,65,66]. Since implant survival depends on many variables [67], it is not possible to attribute outcomes unequivocally to the primary therapy from a scientific perspective. Augmentation performed before implantation, for example, may prevent any inference regarding ridge preservation when survival rates alone are considered. A meta-analysis examined, among other aspects, the necessity of augmentation after ridge preservation [68]. Additional augmentation was required in 9.9% of cases following ridge preservation and in 20.8% after physiological healing. It was emphasized that one study [69], in which more than 50% of participants in the control group required augmentation, was largely responsible for the elevated proportion [68]. A more recent study described a similar trend, reporting that 35.7% of sockets without ridge preservation required additional augmentation, compared with 6.3% in the test group [70]. If augmentation is performed prior to implantation, instead of a one-timed approach, this may also distort results when determining implant position.

Other investigations compare therapies based on subsequent implant success to consider multiple factors simultaneously. In such approaches, results from various clinical and radiological parameters are combined into a single value. If an implant fails in one of the assessed categories, the implantation is classified as unsuccessful. Success rates of the test groups are then compared as percentages [60,62,64,70,71]. This provides a comprehensive overview. In contrast, the advantage of our method lies in the fact that only one examination is required and can be evaluated directly.

The studies mentioned before predominantly compare ridge preservation with the absence of treatment. No study was identified that examined our specific materials in detail after implantation and compared them directly. When other methodologies are considered, the literature, similar to our findings, indicates an advantage of combining a bone substitute material with a blood concentrate. One study investigated comparable materials but did not include a control group without preservation [72]. Cone beam computed tomography images of posterior teeth were analyzed using a measurement protocol first introduced by Jung et al. 2013 [73] and subsequently applied in numerous studies [73,74,75,76,77,78,79,80,81]. Measurement of alveolar ridge width, including more detailed approaches [47], may be complicated by artifacts caused by implants [82,83,84]. Nevertheless, in contrast to bone density measurements, radiographs remain assessable. This approach could therefore also be applied in our context.

In the study of De Angelis et al., L-PRF exhibited the greatest horizontal bone loss, the Bio-Oss Collagen^®^ group showed the second lowest reduction, while the mixed material group demonstrated the least loss [72]. The differences, however, were not statistically significant. This is in accordance with a meta-analysis conducted in 2021. Among the 12 evaluated studies, various methodologies were applied, and overall, the combination of PRF and a bone substitute material achieved the best outcome, followed by bone substitute material alone and finally PRF alone [85]. When these results are compared with our assessment of implant position, the combination group also performed best, consistent with the study of De Angelis et al. [72] and the meta-analysis of Alrayyes et al. [85]. Numerous investigations confirm the positive effect of biologization with autologous blood concentrates. So, it was described that clinically, the combination of FDBA and A-PRF resulted in the least bone loss compared with the use of either material alone. Histological analyses revealed denser bone, more newly formed bone, and less residual material [86,87,88]. Further, beneficial side effects, like reduced pain perception, have been documented with PRF application, including in combination with a bone substitute material [74,89,90]. Reduced postoperative swelling has also been demonstrated for combination therapy compared with bone substitute material alone [80] or PRF alone [89]. Regarding soft tissue healing, adjunctive PRF improved wound healing [74], and radiologically, the combination demonstrated denser bone [74,80,91] with less horizontal and vertical resorption [92].

Overall, a discrepancy becomes apparent between the here-reported implant position results and the above-mentioned studies when the remaining groups are considered. In our analysis, PRF alone yielded better outcomes than the BSM group with Bio-Oss Collagen^®^ alone. In contrast, the literature reports superior results for bone substitute material compared with a blood concentrate [72,78,85]. A possible explanation might be in the distribution of tooth types in our trial. Fewer premolars could be included in the PRF group than in the BSM group. However, the alveolar ridge is reported to be narrower in the premolar region than in the molar region [93,94], and it seems reasonable to assume that inclusion of more molars would more frequently result in a favorable implant position due to the generally greater bone volume or the residual interradicular septum, both adding to available bone structures necessary for implant stabilization. That this might be true is indicated by our sub-analysis of distribution according to tooth type, showing better results for the molar than for the premolar cases in the BSM group. On the other hand, the combination group included the highest number of premolars and nevertheless achieved the best outcome, and sub-analysis, even of the premolar cases, showed often less deviation from the central position. It is therefore not possible to propose a final conclusion that inclusion of fewer premolars necessarily results in improved outcomes.

Nevertheless, it is noteworthy that the same groups showed different outcomes when stratified by tooth type. To the best of our knowledge, to date, no randomized controlled trial or meta-analysis has examined the interaction between treatment group and tooth type as a within-study factor. While differences in post-extraction bone resorption patterns between the maxilla and mandible, and between different tooth types, have been documented in the literature [95,96], no study has systematically examined whether the efficacy of different ridge preservation treatments varies depending on the jaw or tooth type. 

The influence of the latter, and therefore possibly the tooth position per se, on the effectiveness of ridge preservation remains unknown. It is valid to propose that this, possibly related to different resorption rates at the respective position, may have an influence on the efficacy of ridge preservation, and that outcomes therefore differ between molar and premolar sites depending on the treatment applied.

Multiple factors influence successful implant positioning. Bone density, among others, plays a decisive role [54,55] in achieving sufficient primary stability [53]. It is conceivable that although PRF may be associated with less bone preservation capacity, the resulting bone density might be higher, thereby permitting more favorable implant placement. No study was identified that directly compares bone density between a blood concentrate and a bone substitute material. Most investigations compare each test group separately with a control group without preservation using radiological [46,49,50,97] or histological methods [59,75]. Both materials, bone substitute materials and autologous blood concentrates, demonstrate better outcomes than no preservation in a variety of published trials [46,49,50,59,75,97]. The direct difference between the treatment modalities themselves, therefore, remains unclear. A general superiority over no preservation was also confirmed in our analysis.

The advantage of PRF over a bone substitute material may be explained by the fact that PRF is an autologous biological preparation [25] with a specific structural composition [25,28,29] and contains numerous factors [26,27] that can positively influence wound healing. Compared with a bone substitute material, PRF may promote a more natural and physiological healing of the alveolar ridge, which subsequently allows more favorable implant placement. In addition, residual particles of bone substitute materials remain detectable even after more than three months [98,99,100,101]. For example, for BSM Bio-Oss^®^, proportions of up to 42.5% have been reported [100]. These remaining particles may subjectively influence the clinician during surgery when selecting the implant position. To date, residual particles have primarily been described histologically, and remaining Bio-Oss Collagen^®^ particles, as was used here as well, have been shown to be surrounded by newly formed bone [102,103], bone-like tissue [101], or connective tissue [103]. Clinical perception of these particles is therefore unlikely, yet an influence cannot be excluded. Differences might also be detected through tactile feedback. As an autologous material [25], PRF contains no particles, and such an effect can therefore be excluded, which may represent a distinct advantage and might also explain the differing results. Nevertheless, the proposed influence of tactile feedback generated by residual biomaterial particles remains a hypothesis that was neither investigated nor assessed within the present study. Whether this hypothesis holds validity would need to be explicitly examined in a subsequent study specifically designed or adapted to address this question.

Even though each, if not all, of the aforementioned regeneration-related factors may contribute to the observed positional deviations, it has to be kept in mind that the final implant position is likely the result of a complex interplay of known variables, including surgeon experience and preferences, the use and design of surgical guides, implant system design, the planned prosthetics, and patient-specific anatomical conditions such as jaw morphology, bone density distribution, and alveolar ridge geometry [104,105,106,107,108,109].

The present study does not systematically control for or stratify by these variables, which is a limitation of this trial and may represent confounders in the interpretation of positional deviations, and the observed shifts can therefore probably not be attributed only to preservation-related bone remodeling. However, since all implant procedures within this trial were performed by a defined group of experienced surgeons, the influence of inter-operator variability on comparison between the groups is probably low. While the descriptive nature of this pilot study and the absence of systematic control for surgical and anatomical confounders preclude definitive conclusions, the data provide a meaningful basis for the hypothesis that ridge preservation efficacy constitutes a determinant of implant positioning outcomes. Nevertheless, to exclude or minimize any such influence, prospective studies with larger sample sizes, standardized surgical protocols, e.g., in terms of defined guide systems, and, if feasible, stratification by surgeon experience are warranted to validate these preliminary observations and to establish implant position as a reliable readout of ridge preservation effectiveness.

As was noted before, the proposed grid-based assessment of implant position is primarily intended as a systematic evaluation tool for clinical research. Interrater reliability analysis revealed good to very good agreement in two of the three planes (frontal plane: κ = 0.61, *p* < 0.001; horizontal plane: κ = 0.91, *p* < 0.001). In contrast, the sagittal plane evaluation of the inexperienced rater showed less match to the experienced rater with only fair reliability (κ = 0.26, *p* = 0.105). This finding confirms that these assessments currently warrant experienced personnel. With further refinement of the method, more systematic guidelines, e.g., defined distances and angles to neighboring structures, could improve it and allow for reproducibly of the data, independent of raters’ experience.

Taken together, the present study aimed to evaluate whether the type of ridge preservation procedure performed influences the three-dimensional positioning of the implant relative to the original tooth position. Implant position was categorized using the described grid system centered on the former tooth axis: an occlusal 3 × 3 quadrant grid, as well as a 3 × 1 grid in the bucco-lingual and a 3 × 1 grid in the mesio-distal plane, with the central field in each dimension corresponding to the original tooth position.

Based on our results, the observed positional shifts are hypothesized to reflect preservation-dependent alterations of alveolar ridge morphology and the degree of osseous regeneration achieved, both of which may preclude stable implant anchorage at the original tooth axis and necessitate a positional adaptation by the surgeon. Against this background, the findings of the present pilot study provide preliminary support for the proposed hypothesis that implant position is potentially dependent on the efficacy of the selected ridge preservation procedure with respect to the outcomes of bone preservation and regeneration. This suggests that the type and effectiveness of the preservation procedure may indeed influence the available bone volume and quality at the time of implant placement, and consequently, the achievable implant position relative to the original tooth axis.

## 5. Conclusions

Remodeling and resorption processes of the alveolar ridge following tooth extraction may complicate subsequent implantation if it results in insufficient bone volume and density. In our trial, ridge preservation with BSM, PRF, or a combination of both more frequently resulted in optimal implant positions than the absence of treatment. Biologization of the xenogeneic BSM with PRF produced the most favorable outcome, followed by PRF alone and finally BSM. It is important to keep in mind the exploratory and descriptive nature of the here-described preliminary evaluation. Therefore, a definite conclusion about the superiority in effectiveness of any of the groups per se needs to be proven by statistical analysis of the main trials’ endpoints.

Comparison of our observation with other studies confirms the advantage of ridge preservation over physiological healing. In these investigations as well, combination therapy tended to perform best. However, in contrast to our findings, the literature generally reports better outcomes for bone substitute material than for PRF alone. Nevertheless, the here-described novel method using the final implant position for evaluation of ridge preservation efficiency does not focus on radiological, histological, or combined evaluation of the bone regeneration, but includes the definite in vivo situation the clinician is presented with during implantation surgery. The latter, highly influencing the implant positioning as the best achievable primary stabilization, should be achieved to provide the best conditions for long-term implant survival.

Further research is required to investigate these aspects in consideration with the newly established methodology, and to prospectively evaluate the underlying hypothesis that the implant positioning can be a readout for the effectiveness of ridge preservation procedures and their effectiveness on bone preservation and regeneration. In general, the method is straightforward, rapid, and intuitive to apply and provides a relevant assessment of therapeutic success in clinical research.

## Data Availability

Data is contained within the article.

## Abbreviations

The following abbreviations are used in this manuscript:

PRF	Platelet-Rich Fibrin
BSM	Bone Substitute Material
ROI	Region Of Interest
CBCT	Cone Beam Computed Tomography
DICOM	Digital Imaging and Communications in Medicine
